# Tunable Non-linear Refraction Properties and Ultrafast Excited State Dynamics of Dicyanomethylene Dihydrofuran Derivative

**DOI:** 10.3389/fchem.2020.522974

**Published:** 2020-12-03

**Authors:** Linpo Yang, Zhongguo Li, Taihui Wei, Liming Zhou, Feng Li, Shaoming Fang, Yinglin Song

**Affiliations:** ^1^Department of Physics, Harbin Institute of Technology, Harbin, China; ^2^School of Electronic and Information Engineering, Changshu Institute of Technology, Changshu, China; ^3^School of Materials and Chemical Engineering, Zhengzhou University of Light Industry, Zhengzhou, China; ^4^College of Physics, Optoelectronics and Energy, Soochow University, Suzhou, China

**Keywords:** transient absorption spectra, ultrafast dynamic process, non-linear refractive index, intramolecular charge transfer, fluorescence inactivation

## Abstract

The third order non-linear optical response of a dicyanomethylene dihydrofuran compound (DCDHF-2V) was investigated using a Z-scan technique in picosecond and nanosecond time regimes. The results show that DCDHF-2V has excellent excited state non-linear refraction properties on both time regimes, and the non-linear refraction index is also solvent-dependent in the nanosecond regime. The excited state relaxation dynamics of DCDHF-2V were demystified via femtosecond transient absorption spectroscopy. The TA spectra reveal that the solvent viscosities have a substantial impact on the excited state relaxation of DCDHF-2V. The exotic photophysical phenomena in DCDHF-2V reported herein can shed new light on future development of small organic non-linear optical materials with large non-linear coefficients and fast response.

## Introduction

Conjugated organic molecules have attracted much attention in the field of non-linear optics due to their large non-linear coefficients, ultrafast response rates, large damage thresholds, easy processing, and significant improvement in molecular optical properties through simple substituent changes (Gu et al., 2016; Wu et al., 2017a; Wei et al., 2020). The intriguing non-linear optical properties of an organic compound are correlated to its conjugated molecular structure (Kim and Cho, 2009; Xiao et al., 2016; Sadowski et al., 2017; Wu et al., 2017b). Hence, it is of great importance to establish the relationship between molecular structure and non-linear optical properties. It is well-known that the fluorescence properties of conjugated organic molecules (excitation and emission wavelength, intensity, lifetime, etc.) are strongly dependent on the structure and relaxation processes of the molecular excitation state (Suhina et al., 2015, 2016; Ahn et al., 2019; Li et al., 2019). The fluorescence properties can be fine-tuned by changing of the polarity, viscosity, and pressure of the environment (Dreger et al., 1992, 1998; Willets et al., 2004, 2005). The non-linear absorption and refraction properties of organic molecules are closely related to the absorption cross-section and refraction volume of excited states. The excited state properties are influenced by changes in the physical properties of the solvent. However, the research of solvent effects on organic molecules non-linear optical (NLO) properties is still scarce (Machado et al., 2016). Therefore, it is highly interesting to study the impact of the environment on the third order NLO response of an organic molecule.

In the D-π-A molecular system, charge transfers from the donor group to the acceptor group under light excitation. This process is called intramolecular charge transfer (ICT) (Lim et al., 2017). If the donor and acceptor groups are connected by a rotatable single bond, the donor and acceptor groups rotate around the single bond due to the strong photoinduced ICT after being excited by photons. At this time, the donor and acceptor groups are in an orthogonal state, the conjugate structure of the molecule is destroyed, and a twisted ICT (TICT) (Teran et al., 2016) excited state is formed. In the TICT process, the torsion of the molecules is greatly affected by the solvent viscosity and external pressure. To understand the influence of the environment on the fluorescence properties of an organic conjugated system, it is important to analyze the decay pathways of the excited states (radiation transition, intersystem transition, energy transfer, etc.) (Makhal et al., 2016; Jia et al., 2018). Among various organic receptor units, dicyanomethylene dihydrofuran (DCDHF) has received considerable research interest in recent years due to its strong electron absorption ability (Lu et al., 2009; Hao et al., 2017) and single molecule fluorescence property (Lord et al., 2007, 2009; Lu et al., 2009). Previous literature reports that the fluorescence intensity of DCDHF derivative molecules with different donor groups is heavily dependent on solvent viscosity and external pressure, and the reasons for its fluorescence inactivation have also been widely discussed (Grabowski et al., 2003; Willets et al., 2004; Zhang et al., 2012; Park et al., 2013). By means of experimental tests and a series of electronic structure calculations, Katherine et al. understand in-depth that the distortion of some of the chemical bonds in the DCDHF molecule can affect the process of radiation transition and non-radiative transition (Willets et al., 2004) so that the fluorescence quantum yield in the solution can be improved by appropriate modification to suppress the internal rotation movement of the fluorophores. Font-Sanchis et al. (2010) synthesized the DCDHF copolymer of dicyandiamide-containing units, and the experimental results show that the reaction of the material is closely related to the glass transition temperature, but the change of temperature does not affect the refractive index of the material. Suhina et al. (2016) synthesized rigid fluorescent molecules (DCDHF-derived compounds 1 and 2), confirming the existence of a fast, non-radiative relaxation pathway due to the excited state—the rotation of a specific bond in the molecule, so in the fluorescent molecule, only weak fluorescence can be emitted in low-viscosity solutions, and strong fluorescence can be observed in very viscous media. The team then proved that the fluorescence intensity of fluorescent molecules containing DCDHF motif increased with the increase of solvent viscosity, and single and double bond rotation were the non-radiative decay pathways of this molecule. These results demonstrate that the photophysical properties of DCDHF are sensitive to the surrounding media.

DCDHF derivative molecules show good NLO properties. Han et al. (2008a,b) synthesized the DCDHF-2V molecule and studied the second order NLO properties of DCDHF-2V/PMMA film. The second-order non-linear coefficient d_33_ was 15.2 pm/V at wavelength of 1,064 nm. Tang et al. (2016a,b) synthesized a series of DCDHF derivatives with different substituent groups. They find that the different substituent groups and conjugate length can significantly influence the second order polarizability of DCDHF derivatives. DCDHF dyes exhibit two-photon absorption (TPA) characteristics in the near-red region, which can be used in two-photon fluorescence imaging (Schuck et al., 2005). He et al. (2011) prove that the twisted π-system chromophores can significantly enhance the non-linear refraction coefficient due to the twisted structure. These studies show that DCDHF derivatives with twisted structures have excellent NLO properties, especially the large non-linear refraction coefficient. The relationship between excited state dynamics of DCDHF dyes and the NLO properties is still unclear.

In this work, we study the impact of solvent viscosity on the non-linear refractive index of a DCDHF derivative (DCDHF-2V). The synthesized molecule was studied by using UV-vis, fluorescence spectra, and Z-scan technology. The ultrafast excited-state decay processes of DCDHF-2V were investigated via femtosecond transient absorption spectroscopy. Our results could shed new light on the development of novel organic NLO materials.

## Synthesis of DCDHF-2V and the Experimental Method

### Synthesis of DCDHF-2V

**Figure 1:**
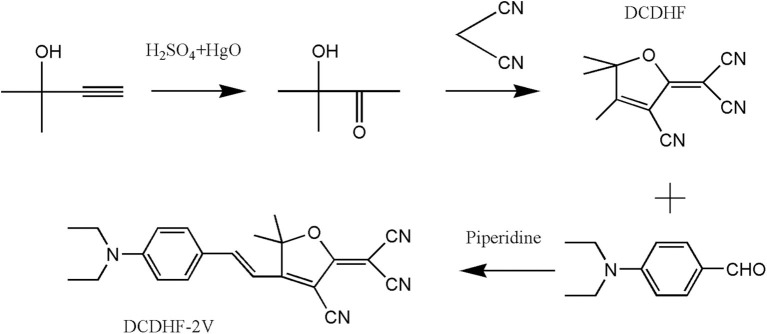
Structural formula and synthetic pathway of molecular DCDHF-2V.

The sample synthesis method is referred to Ito et al. (2013), and the synthesis processes are shown in Figure 1. (1) Concentrated sulfuric acid (1.9 mL) was added dropwise to deionized water (10 mL) followed by mercury oxide (1.3 g, 6 mmol). Then, 2-methyl-3-butyn-2-ol (8.4 g, 100 mmol) was added dropwise to the previous solution under an ice bath, slowly heated to 70°C, and stirred for 1 h. The sample was fractionated, and a fraction at 140°C was collected, and then, the product Z (3-Hydroxy-3-methyl-2-butanone) was obtained. (2) A mixture of sodium hydride (0.05 g, 7 mmol) and absolute ethanol (1 mL) was added to a mixture of Z (2.05 g, 20 mmol) and malononitrile (2.64 g, 40 mmol) in THF (30 mL). The resulting solution was refluxed at a water bath temperature of 50°C for 12 h. Then, it was steamed (45°C) to remove tetrahydrofuran, followed by recrystallization from ethanol to obtain the product DCDHF. (3) In a round-bottom flask, THF (20 mL) for 4-Diethylaminobenzaldehyde (2.13 g, 12 mmol), DCDHF (1.98 g, 10 mmol), piperidine 5 drops) was added in turn, and the mixture was refluxed for 14 h in a dark environment. Then, it was concentrated by rotary evaporation and diluted with ethyl acetate, and the organic layer was washed with hydrochloric acid (0.1 mol/L), dried and concentrated, and then recrystallized from anhydrous ethanol to obtain compound DCDHF-2V. ^1^H NMR (600 MHz, DMSO) δ 7.91 (d, J = 15.7 Hz, 1H), 7.75 (d, J = 8.9 Hz, 2H), 6.88–6.78 (m, 3H), 3.51 (m, 4H), 1.75 (s, 6H), 1.16 (t, 6H). ^13^CNMR (151 MHz, DMSO) δ 177.29, 175.34, 151.84, 149.34, 133.30, 121.55, 113.51, 112.68, 112.05, 112.02, 107.86, 98.00, 91.43, 50.61, 44.29, 25.66, 12.48.

### Experimental Method

The UV-vis spectroscopy of the sample was carried out using a UV-visible spectrometer with model Hitachi U-3900H (made in Japan). Fluorescence spectroscopy was performed using a steady-state fluorescence spectrometer with model FLS980 (made in the UK). During the test, the sample (concentration 5 × 10^−7^ mol/ L) was placed in a 10-mm-thick quartz colorimetric dish. All tests were performed at room temperature without special instructions.

**Figure 2:**
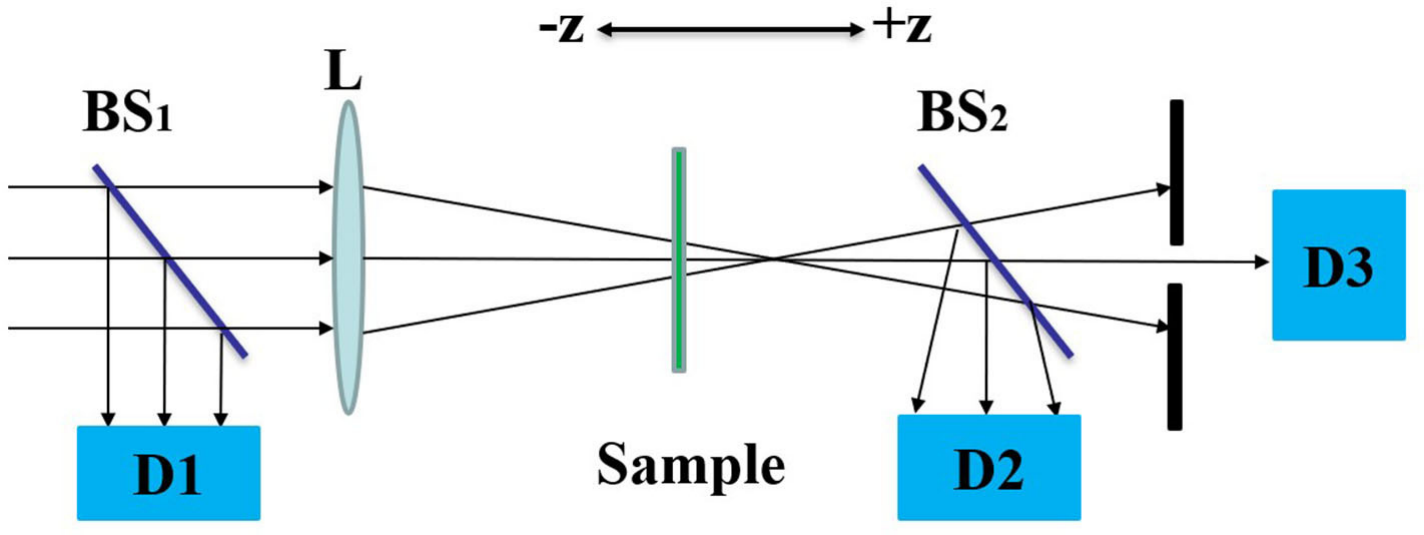
The optical path of Z-scan. Where **BS** are beam splitters, **L** is optical lens and **D** are detectors.

Figure 2 shows the Z-can experimental optical path. The sample is placed near the focal point of lens **L** and is controlled to move around the focal point along the Z-axis by a stepper motor. The input light intensity will change with the position of the sample. During the movement of the sample, the laser pulse energy through the sample is detected by **D2**. The open-aperture Z-scan curve corresponds to the non-linear absorption of the sample. By placing a small hole in front of **D3**, the laser pulse energy at the center of the spot is obtained, which is the closed-aperture Z-scan experimental curve, and the non-linear refraction properties of the sample are obtained. In the Z-scan experiment, a semiconductor pumped solid-state laser with laser model GKPPL-1064-1-20 (produced by Beijing Guoke Laser Technology Co., Ltd.) was used as the light source. The laser pulse width and repetition rate were 15 ps and 10 Hz, respectively. In this experiment, the laser wavelength was λ = 532 nm, the focal length was 0.4 m, and the focal spot radius was 30 μm. The DCDHF-2V sample was dissolved in a methanol solution in which the solution concentration was 4.5 × 10^−6^ mol/L, the sample cell thickness was 2 mm, and the linear transmittance to the 532 nm laser was 0.6. Before testing the samples, we performed a Z-scan test on the methanol solution at the same incident light energy. No non-linear absorption and refraction were observed, so the effect of the solvent on the experimental results was excluded.

The transient absorption spectrum was measured by an ultrafast transient absorption spectrometer (HARPIA-TA, LIGHT CONVERSION). In the transient absorption spectra experiment, the pump light has a wavelength of 400 nm with a pulse width of 190 fs and repetition frequency of 6 kHz. The probe light is generated by focusing a 1,030-nm laser on a non-linear crystal.

## Results and Discussion

### UV–Visible Absorption and Fluorescence Spectra

**Figure 3:**
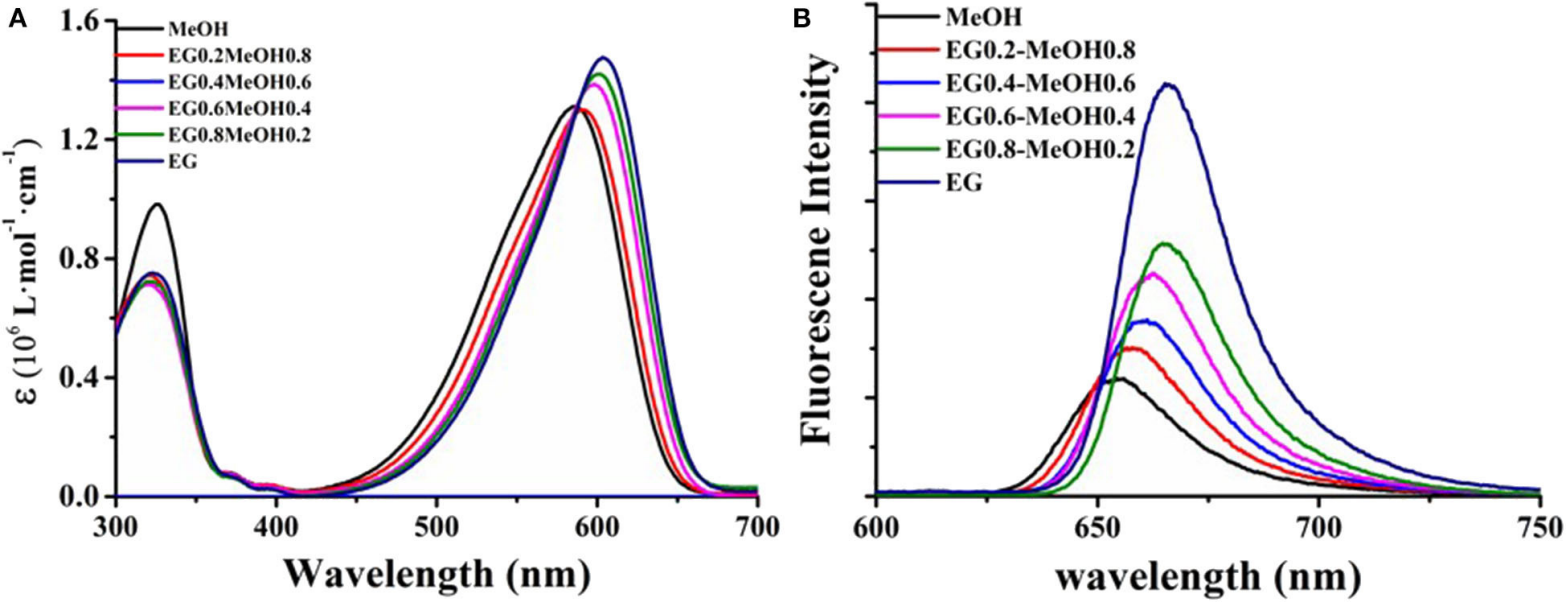
UV-vis absorption spectra **(A)** of DCDHF-2V in different ratios of methanol and ethylene glycol solution and fluorescence spectra **(B)** at 500 nm excitation.

The UV-vis absorption spectra of the samples in different solvents and the fluorescence emission spectra excited by a 500-nm wavelength are shown in Figure 3, in which the UV-vis spectra were normalized at the position of the strongest absorption peak, and the fluorescence intensity of the samples in different solvents is the actual intensity at the same concentration. In Figure 3A, it can be seen that there are two absorption peaks in the UV-visible region of compound DCDHF-2V in methanol solution, corresponding to 325 and 586 nm, respectively, and the absorption intensity is the highest at 586 nm. When the proportion of ethylene glycol in the solvent increases, the position of the absorption peak at 325 nm does not change, and the absorption peak at 586 nm appears red-shifted. When the solvent is completely ethylene glycol, the absorption peak at 586 nm is red-shifted to 604 nm.

As shown in Figure 3B, the fluorescence emission peak of the sample excited by the 500-nm wavelength is located at 658 nm and at the edge of the resonance absorption peak of the sample, which satisfies the rule of the transition from the bottom excited state to the ground state. When the content of ethylene glycol in the solvent increases, the red shift of the fluorescence emission peak of the sample is related to the change of the polarity of the solution (consistent with the ultraviolet-visible absorption spectrum), and the increase of the fluorescence intensity is mainly related to the viscosity of the solvent. It can be seen from the UV-vis spectrum that the absorption at 500 nm decreases with the increase of ethylene glycol ratio at the same concentration. At this time, the fluorescence intensity of DCDHF-2V still increases with the increase of solvent viscosity, indicating that the sample shows stronger fluorescence quantum yield in high-viscosity solvent, which is consistent with the report of the same kind of molecules (Suhina et al., 2016; Qian et al., 2017).

### NLO Properties of DCDHF-2V

**Figure 4:**
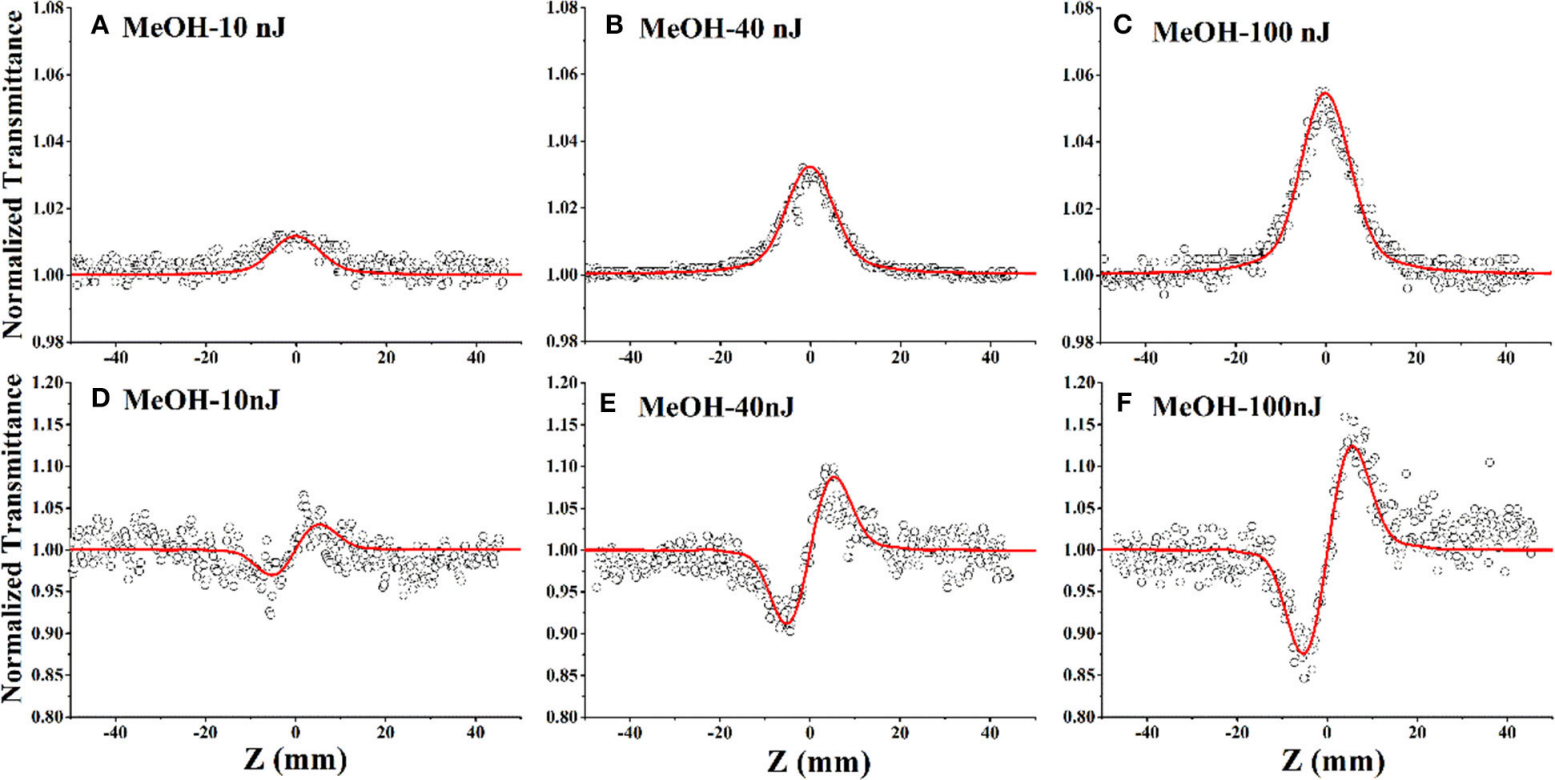
Open-aperture **(A–C)** and closed-aperture **(D–F)** Z-scan test results for DCDHF-2V methanol solution at different energies under 15 ps, 532 nm pulse laser (the hollow scatter are experimental data and the solid lines are the fitting lines).

To study the NLO properties of the DCDHF-2V molecules, we tested the sample solution by Z-scan measurement. Because the sample may have both non-linear absorption and non-linear refraction, the closed-aperture Z-scan experimental results need to be divided by the open-aperture experimental results to eliminate the influence of the non-linear absorption effect on the non-linear refraction experimental results; the Z-scan curves are shown in Figure 4.

The open-aperture Z-scan tests in Figures 4A–C shows that the sample transmittance increases with the increase of incident light intensity; that is, the compound DCDHF-2V exhibits saturated absorption under laser excitation at the 532-nm wavelength. According to the previous UV-vis absorption spectrum (Figure 3), it can be seen that the 532-nm wavelength laser is located near the resonance absorption peak of the DCDHF-2V molecule, which indicates that the DCDHF-2V molecule has a strong linear absorption to the incident light, so the saturated absorption of the sample is mainly caused by ground state bleaching (Zieleniewska et al., 2018). Figures 4D–F shows that the normalized closed aperture Z-scan curve shows a valley–peak shape, which indicates that the DCDHF-2V molecule has positive non-linear refraction. The variation of absorption and refraction coefficients with light intensity can be described by the following formula (Sheik-Bahae et al., 1990):

(1) $$\begin{array}{l} \alpha =\alpha_{0}+\beta I\\ n=n_{0}+n_{2}I \end{array}$$

**Figure 5:**
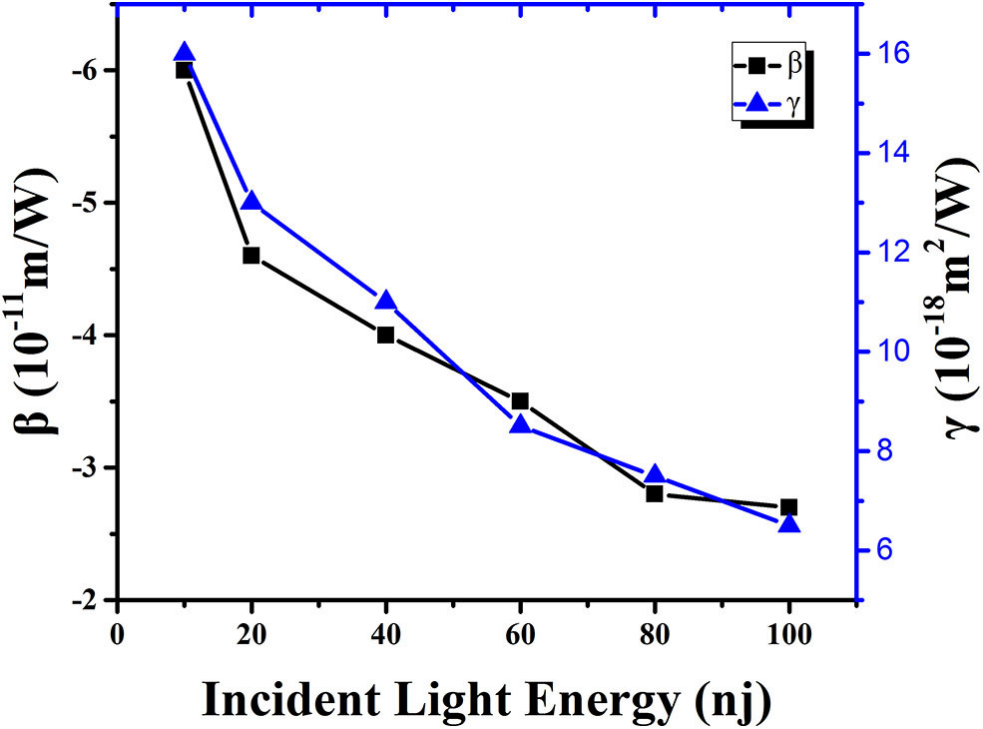
Non-linear absorption coefficient (black solid point) and refractive index (blue solid point) of DCDHF-2V methanol solution as a function of incident light energy under 15 ps, 532 nm pulse laser.

Among them, α_0_, β, *n*_0_, *n*_2_ are the linear absorption coefficient, the third-order effective non-linear absorption coefficient, the refractive index, and the non-linear refractive index of the sample, respectively. When the light intensity is relatively large, the absorption and refractive index of the sample change with the change of light intensity, which causes the sample absorption and refractive index to change and finally leads to non-linear absorption and refraction effects. The data measured by open and closed-aperture Z-scan are fitted, respectively, and the third order non-linear optical coefficients β, *n*_2_ of the sample are obtained (shown in Table 1 and Figure 5). The detailed fitting procedure can be found in Sheik-Bahae et al. (1990).

**Table 1:** Non-linear absorption and refraction coefficients of DCDHF-2V in methanol solution under 15 ps, 532 nm pulse laser.

**Incident light** **energy (nJ)**	**Incident light** **intensity (10^**12**^ W/m^**2**^)**	***α*_2_** **(10^−11^ m/W)**	***n*_2_** **(10^−18^ m^2^/W)**
10	0.64	−6.0	16.0
20	1.28	−4.6	13.0
40	2.55	−4.0	11.0
60	3.83	−3.5	8.5
80	5.10	−2.8	7.5
100	6.38	−2.7	6.5

**Figure 6:**
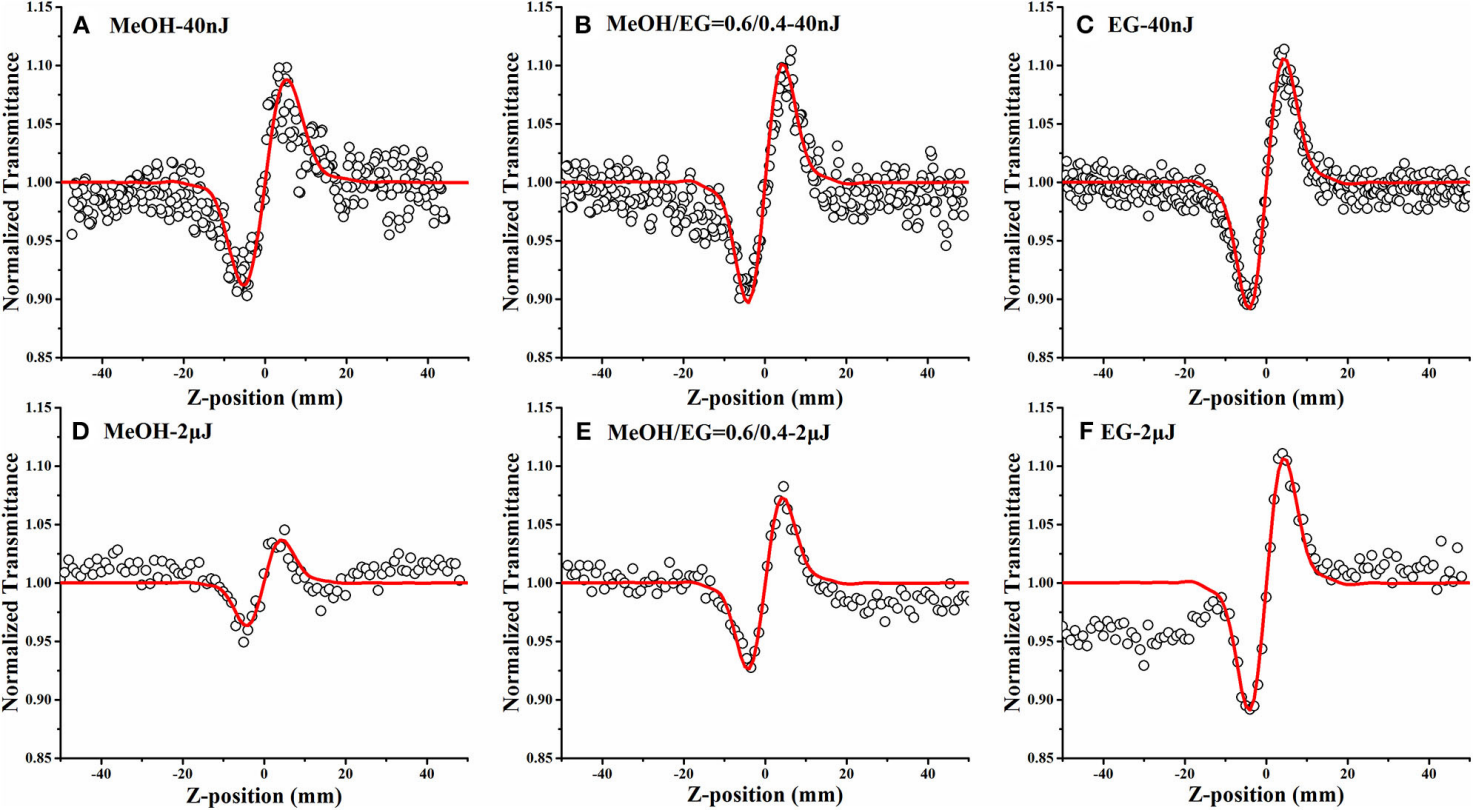
Closed-aperture Z-scan results of DCDHF-2V in different MEOH/EG ratio solutions at 15 ps **(A–C)** and 4 ns **(D–F)** pulse width lasers (the hollow dots are the experimental data, and the red solid lines are the fitting curve).

By observing the data in Table 1 and the trend of the curve in Figure 6, it is obvious that the non-linear absorption coefficient (β) of the sample shows a decreasing trend with the increase of incident light energy. When the energy is 10 nJ, the non-linear absorption coefficient of the sample in the methanol solution is 6 × 10^−11^ m/W. When the incident light energy is increased to 100 nJ, the non-linear absorption coefficient of the sample in methanol solution is reduced to 2.7 × 10^−11^ m/W. This result indicates that the origin of NLO response of the DCDHF-2V molecule is an excited-state mechanism rather than an instantaneous bound-electronic mechanism (Niu et al., 2018; Wen et al., 2018).

We simplify the energy level model to a two-level model consisting of only the ground state and the first excited state, ignoring the relaxation processes in the excited states. The change in the number of ground state and excited state molecules can be simply described by a two-level model with rate Equation (2), and the light intensity transmitted through the sample can be obtained by Equation (3):

(2) $$\{\begin{array}{l} \frac{dN_{0}}{dt}=-\frac{\sigma_{0}N_{0}I}{\hbar \omega }+\frac{N_{1}}{\tau }\\ \frac{dN_{1}}{dt}=\frac{\sigma_{0}N_{0}I}{\hbar \omega }-\frac{N_{1}}{\tau }\\ N_{0}+N_{1}=N \end{array}$$

(3) $$\begin{array}{l} \frac{dI}{dz}=-\left(\sigma_{0}N_{0}+\sigma_{1}N_{1}\right)I\\ \;=-\left[\sigma_{0}N+\left(\sigma_{1}-\sigma_{0}\right)N_{1}\right]I\\ \;=-\left(\alpha_{0}+\beta I\right)I \end{array}$$

*N*_0_ is the number of ground state molecules, *N*_1_ is the locally excited state molecule number, σ_0_ is the molecular linear (ground state) absorption cross-section, τ is the lifetime of the first excited state, and *N* is the molecular number of the sample in the unit volume solution. The lifetime of the first excited state is generally much larger than the pulse width in the experiment, so the relaxation process of the excited state to the ground state can be ignored in the discussion. Therefore, the light intensity through the sample is only related to the incident light intensity and the difference of absorption cross-section between the first excited state and the ground state. With the increasing of light intensity, the decrease in *N*_0_ makes the further excitation of the samples more difficult. The non-linear absorption coefficient β under specific light intensity reflects the number of excited state molecules *N*_1_. Similarly, the third-order non-linear refraction system of the sample shows the same trend as the non-linear absorption coefficient with the change of light intensity, which indicates that the non-linear refraction of DCDHF-2V is mainly caused by the difference between the refractive volume between the excited state and the ground state of the molecule. Therefore, the non-linear refraction of DCDHF-2V molecules is closely related to the excited states refraction.

### NLO Properties Depending on Solvent and Laser Pulse Width

The twist and relaxation processes of the DCDHF-2V molecular excited structure are dependent on the viscosity of solution. Hence, studying the relationship between the non-linear refractive index of DCDHF-2V and the viscosity of the solvent is of great help to reveal the origin of non-linear refraction of DCDHF-2V. The closed-aperture Z-scan tests of DCDHF-2V in different MEOH/EG solvent solutions under 15 ps and 4 ns pulse width lasers were carried out, and the experimental results are shown in Figure 6. All closed-aperture Z-scan results are divided by the opening results to eliminate the energy changes caused by non-linear absorption. During the test, the concentration of solution and the energy of incident light are guaranteed to remain unchanged. The thermal aspects interfere with the non-linear absorption and refraction due to the strong linear absorption at 532 nm. Thermal aspects usually occur under nanosecond laser pulse due to the larger pulse energy and longer pulse duration. In our experiment, we reduced the thermal aspects on the Z-scan by reducing the laser pulse energy <2 μJ and increasing the sample transmittance more than 60%. In general, the thermal aspects cause self-defocusing and reverse saturation absorption (Yang and Song, 2009). DCDHF-2V showed self-focusing and saturation absorption. Therefore, the thermal aspects of NLO properties can be ignored in our experiments.

The data were fitted to the parameters of the fitted curves to obtain the non-linear refractive indexes of DCDHF-2V under different viscosity solvents as shown in Table 2. It can be seen that, under the excitation of the 15 ps pulse width laser, the non-linear refractive index of the DCDHF-2V molecule hardly changes with the viscosity of the solvent. However, under the 4 ns pulse width laser excitation, the non-linear refractive index increases with the viscosity of the solution. Under the excitation of the 15 ps pulse laser, the non-linear refraction of DCDHF-2V is mainly determined by the difference between excited state and ground state refraction volume. Under the excitation of the 4 ns pulse laser, the non-linear refractive coefficient of the molecule is also related to the relaxation process of the excited state because of the long pulse width. As described by the rate equation in formula (2), the influence of the relaxation processes of excited state molecules on the distribution of molecules in different states must be considered.

**Table 2:** Non-linear refractive index *n*_2_ of DCDHF-2V in different viscosity solvents (unit: 10^−17^ m^2^/W).

**Solvent**	***n*_2_ (10^−17^ m^2^/W)**	
	**15 ps (40 nj)**	**4 ns (2 uj)**
Methanol	1.1	2
Methanol/Ethanediol = 0.8/0.2	1.1	2.7
Methanol/Ethanediol = 0.6/0.4	1.05	3.4
Methanol/Ethanediol = 0.4/0.6	1.1	4.0
Methanol/Ethanediol = 0.2/0.8	1.1	4.9
Ethanediol	1.1	5.9

### Transient Absorption Spectra and Ultrafast Dynamic Processes

**Figure 7:**
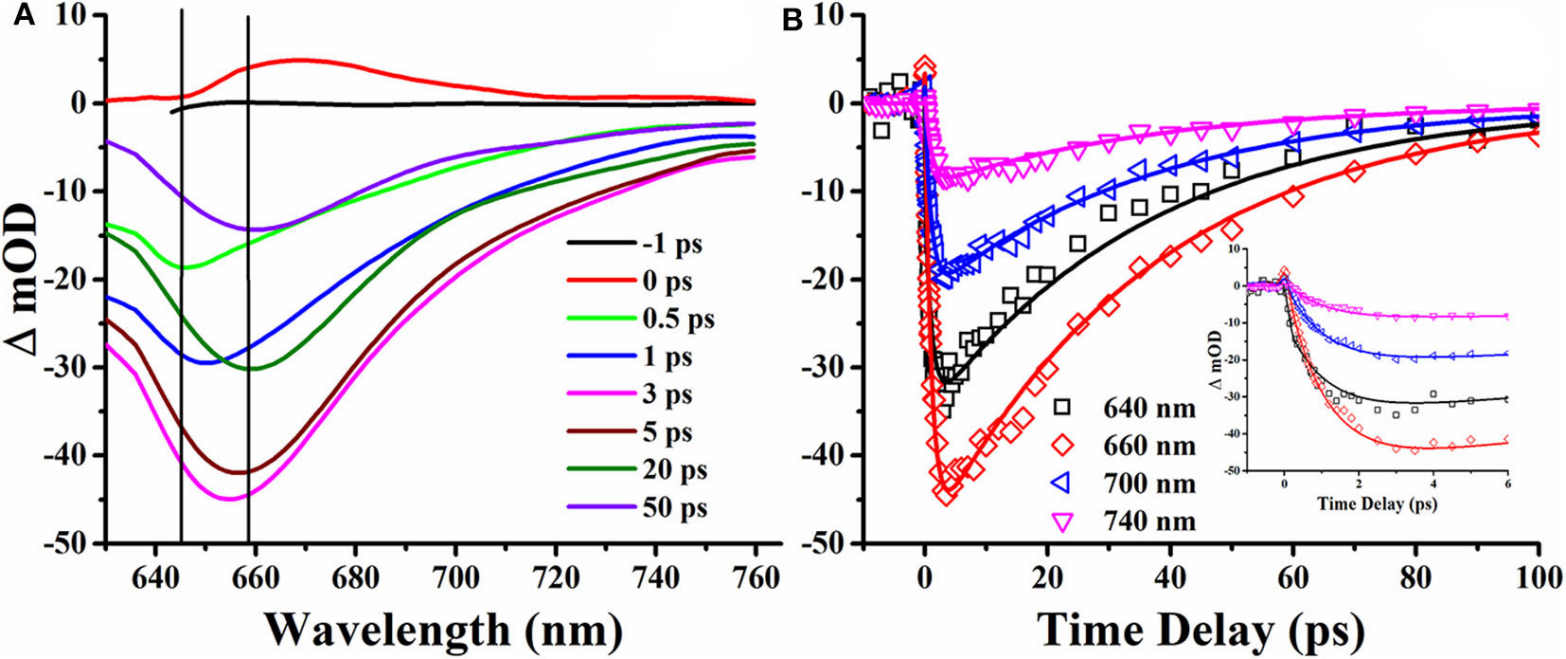
Transient absorption spectrum **(A)** and time delay curve **(B)** of DCDHF-2V methanol solution under 190 fs, 400 nm pulse laser excitation.

To investigate the excitation and relaxation process of DCDHF-2V molecules under photoexcitation in detail, transient absorption spectra of DCDHF-2V methanol solution were measured, and the results are shown in Figure 7. The change in absorption intensity at a specific wavelength obtained by transient absorption spectroscopy experiments is expressed by the change in optical density (ΔOD), calculated as

(4) $$\begin{array}{l} \Delta OD=-\log_{10}\left(T/T_{0}\right) \end{array}$$

*T* is the transmittance of the sample after pumping, and *T*_0_ is the linear transmittance of the sample. It can be seen from Figure 7A that the optical density in the TAS is negative, which shows that the transient absorption of DCDHF-2V is smaller than that of linear absorption. It can be seen from the transient absorption spectrum that the absolute value of the optical density shows a trend of increasing at zero-time delay, which shows excited states absorption and then decreasing at different time delays, and reaching the strongest at 3 ps. The wavelength in TAS is away from the linear absorption region of the DCDHF-2V molecule. The negative optical density indicates the radiative transition processes of DCDHF-2V. In the time delay of 0.5–3 ps, the negative transient absorption peak increased, and the fluorescence peak is red-shifted from 645 to 658 nm, which is identified as the ICT process (Sasaki et al., 2016). The saturation absorption peak observed after the 3-ps time delay in the transient absorption spectrum is consistent with the fluorescence peak of the sample in methanol, showing a radiation transition in the ICT state. A global fitting method was used in fitting the transient delay curves. Three lifetime parameters were obtained: 0.38, 1.04, and 36.8 ps, respectively. Based on the analysis of the transient absorption spectrum, the three lifetimes are relaxed by the singlet excited state relaxation, ICT, and the lifetime of the ICT state, respectively. The fluorescence lifetime of organic small molecules is usually on the order of nanoseconds, and the ultrafast fluorescence process of 36.8 ps of DCDHF-2V in methanol solution stimulates our interest in further exploration. In previous studies, DCDHF derivatives have multiple non-radiative transition processes: TICT and conical intersect (CI) (Grabowski et al., 2003; Font-Sanchis et al., 2010; Zhang et al., 2012; Suhina et al., 2016). In DCDHF-2V, charge transfers from N, N-diethyl aniline to DCDHF fluorophores after being excited by photons. At this same time, the molecule twists itself around the middle olefinic bond due to the Coulomb force.

**Figure 8:**
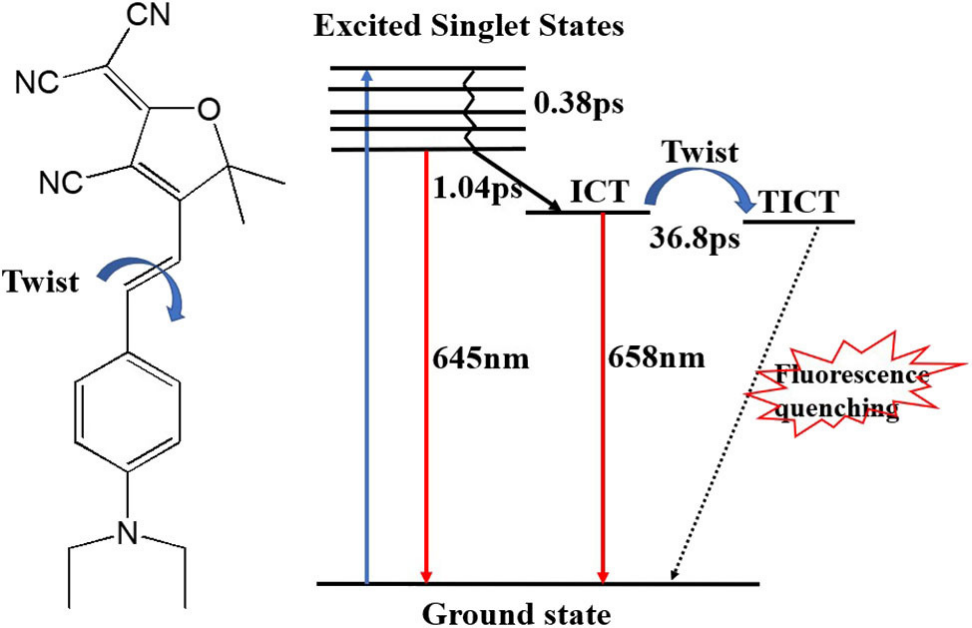
Schematic diagram of the DCDHF-2V excitation and relaxation process.

During the twisting process in DCDHF-2V, the excited state potential energy surface intersects with the ground state, causing a rapid quenching of fluorescence. Therefore, the fluorescence performance of DCDHF-2V molecules increases with the increase of solvent viscosity, which can be attributed to the limiting effect of solvent viscosity on molecular torsion. We describe the excitation and relaxation processes of the DCDHF-2V molecule with an energy level model as shown in Figure 8. The ground state DCDHF-2V molecule is excited to the excited state by absorbing a photon, and then returns to the first singlet excited state after rapid relaxation (0.37 ps). A few molecules in the first singlet excited state return to the ground state through a radiative transition, emitting 645 nm fluorescence. The rest of the molecules relax to the ICT state through the ICT process (1.04 ps). ICT fluorescence contributes a major part of the fluorescence spectrum with a wavelength of 658 nm. The change of the solvent viscosity can improve the fluorescence quantum yield of the DCDHF-2V, which is mainly due to the limitation of molecular rotation of the ICT state, thereby leading to the increase of the fluorescence lifetime and the improvement of the fluorescence efficiency.

For the NLO properties of DCDHF-2V under 15 ps pulse lasers, a large number of molecules are in the ICT state, causing a great non-linear refraction effect. When the viscosity of the solvent increases, the limitation of the solvent viscosity on the molecular torsion extends ICT state lifetime. The number of ICT state molecules changes a little for a duration of 15 ps because of the longer ICT state lifetime. Therefore, the DCDHF-2V molecule non-linear refractive index has little change with the viscosity of the solution under the 15 ps pulse laser. Under the 4 ns pulse laser, the non-linear refractive index of the DCDHF-2 V molecule is related to the increasing of the molecular dipole moment caused by ICT, and the value is mainly affected by the lifetime of the ICT state because of the longer laser pulse duration.

## Conclusion

In this paper, we investigated the effect of solvent viscosity on the photophysical properties of a DCDHF derivative DCDHF-2V. The results show that the molecular fluorescence efficiency increases with the increase of solvent viscosity. Then, the influence of the solvent viscosity on the excited state relaxation process of DCDHF-2V is discussed by the femtosecond transient absorption spectrum, and it was found that the TICT process of the molecule is limited by the increase of the viscosity of the solvent. Interestingly, through the corresponding relationship between the non-linear refractive index and the solvent viscosity of DCDHF-2V molecules at 15 ps and 4 ns pulsed lasers, it is proposed that the non-linear refraction of DCDHF-2V is mainly caused by the molecular ICT state, and the molecular non-linear refraction in the nanosecond time domain range is also affected by the limitation of solvent viscosity on TICT process.

## Data Availability Statement

The datasets generated for this study are available on request to the corresponding author.

## Author Contributions

YS, LY, and SF contributed conception and design of the study. LY, SF, and LZ contributed the synthesis and testing of samples. LY, ZL, YS, TW, and FL organized the database and performed the statistical analysis. LY wrote the first draft of the manuscript. ZL and TW wrote sections of the manuscript. All authors contributed to manuscript revision, read, and approved the submitted version.

## Conflict of Interest

The authors declare that the research was conducted in the absence of any commercial or financial relationships that could be construed as a potential conflict of interest.
